# Increase in Physical Activity After Bariatric Surgery Demonstrates Improvement in Weight Loss and Cardiorespiratory Fitness

**DOI:** 10.1007/s11695-018-3439-x

**Published:** 2018-08-13

**Authors:** Onno M. Tettero, Tianna Aronson, Rens J. Wolf, Malou A. H. Nuijten, Maria T. E. Hopman, Ignace M. C. Janssen

**Affiliations:** 1 Nederlandse Obesitas Kliniek (Dutch Obesity Clinic), Postbus 601, 3700AP Zeist, The Netherlands; 2 Medtronic, Minneapolis, MN USA; 3 Radboud University Medical Center, Nijmegen, The Netherlands

**Keywords:** Bariatric care, Cardiorespiratory fitness, VO2max, Weight loss, Physical activity

## Abstract

**Background:**

Low cardiorespiratory fitness is strongly associated with cardiovascular diseases and mortality. Although increased physical activity can improve cardiorespiratory fitness, this relationship has not been examined in a large bariatric population undergoing perioperative care focusing on long-term lifestyle change.

**Objectives:**

To evaluate changes in physical activity, weight loss, and cardiorespiratory fitness up to 24 months after bariatric surgery, and to evaluate the relationships of change in physical activity with weight loss and change in cardiorespiratory fitness.

**Materials and Methods:**

Four thousand seven hundred eighty-five patients who underwent primary Roux-en-Y gastric bypass or sleeve gastrectomy between January 2012 and December 2014 were included. Physical activity was assessed by the Baecke questionnaire (work, leisure, and sport activity) and cardiorespiratory fitness, defined as VO_2_max relative to fat-free mass (VO_2_max/FFM), was assessed by the Åstrand test.

**Results:**

Twenty-four months postoperative, significant improvements were seen in sport and leisure activity assessments (*n* = 3548, *P* < 0.001), weight loss (*n* = 3695, *P* < 0.001), and VO_2_max/FFM (*n* = 1852, *P* < 0.001). Furthermore, regression analysis showed that change in leisure activity was positively associated with weight loss (*n* = 3535, *ß* = 1.352, *P* < 0.001) and change in sport activity was positively associated with change in VO_2_max/FFM (*n* = 1743, *ß* = 1.730, *P* < 0.001).

**Conclusion:**

Bariatric surgery complemented by a comprehensive bariatric care program can lead to improvement in physical activity, as well as weight loss and improvement in cardiorespiratory fitness. The positive associations of change in leisure activity with weight loss and change in sport activity with cardiorespiratory fitness suggest that bariatric care programs can enhance postoperative outcomes by improving the patient’s physical activity.

## Introduction

Bariatric surgery has emerged as the most effective treatment option for those suffering from morbid obesity [1, 2]. The American Society of Metabolic and Bariatric Surgery (ASMBS), the American Heart Association (AHA), the American Association of Clinical Endocrinologists (AACE), and the National Institute for Health and Care Excellence (NICE) have recommended multicomponent perioperative care that includes increased physical activity for patients undergoing bariatric procedures [3–6].

Research has shown that increased physical activity can improve cardiorespiratory fitness in non-bariatric patients [7]. Cardiorespiratory improvements could result in substantial health benefits since low cardiorespiratory fitness is associated with cardiovascular disease and mortality [8]. Although previous studies have demonstrated associations of increases in physical activity with weight loss and cardiorespiratory fitness after bariatric surgery, these studies only focused on exercise interventions and short-term outcomes [9, 10]. As a result, the relationship between physical activity and cardiorespiratory fitness remains unclear in a bariatric population undergoing perioperative care focusing on long-term lifestyle change.

The aim of this study was to assess changes in physical activity (work, leisure, and sport), weight loss and cardiorespiratory fitness up to 24 months postoperative and to analyze the relationships between change in physical activity and weight loss, and between change in physical activity and change in cardiorespiratory fitness in a large group of bariatric patients undergoing perioperative care focusing on long-term lifestyle change.

## Methods

### Study Population

Twenty-four-month follow-up data was collected retrospectively from patients who underwent primary Roux-en-Y gastric bypass (91%, *n* = 4359) or sleeve gastrectomy (9%, *n* = 426) surgery between January 2012 and December 2014 and combined their procedure with a structured group counseling bariatric care program. Those patients who were unable to integrate into group sessions (e.g., non-Dutch speakers) were offered an individual care program as an alternative and are not included in this study.

### Pre- and Postoperative Care

Eight Dutch bariatric care centers involved in this study delivered identical pre- and postoperative care programs to patients undergoing surgery for morbid obesity. Treatment consisted primarily of a bariatric procedure integrated into an intensive lifestyle change program. The program focused on developing a new healthy lifestyle, of which physical activity is an essential element. Treatment was provided by a multidisciplinary team consisting of a bariatric physician, dietitian, psychologist, physiotherapist, internist, and surgeon. All measurements (e.g., weight, Baecke physical activity questionnaire, Åstrand bicycle tests, and other data) were assessed at the treatment centers.

After screening, patients were enrolled in treatment groups of eight individuals and began the preoperative care program where they participated in group visits on a weekly basis for 7 weeks. The goal of this stage was to educate patients about healthy eating habits, physically active behavior, and to encourage patients to become intrinsically motivated for lifestyle changes. After surgery, patients began the 15-month lifestyle change program, during which patients had group visits every 3 weeks. Group visits throughout both the preoperative and postoperative stage consisted of three consecutive 1-h sessions with a psychologist, dietician, and physiotherapist respectively. The goal of the 15-month lifestyle change program was for the patients to adopt a new independent healthy lifestyle, without the support of the bariatric care team. Additionally, patients had an individual medical check with the bariatric physician at 2 weeks and every 3 months postoperative, up to 15 months.

Although patients were not subjected to a specific physical activity regimen during the program, they were educated about moderate-intensity daily physical activity, aerobic training, and muscle-strengthening according to the recommendations of the World Health Organization 2011 guidelines for physical activity [11]. In addition, patients were coached on SMART (Specific, Measurable, Assignable, Realistic, and Time-related) goal setting and were trained to recognize and cope with body signals like pain, exhaustion, and fatigue [12]. Furthermore, patients were encouraged to pursue individually preferred physical activities to enhance long-term intrinsic motivation.

After the 15-month lifestyle change program, patients visited the clinic annually up to 5 years postoperatively for follow-up measurements, medical health checks, and additional individual patient-specific support if needed. Data were directly uploaded into an electronic medical record using an automated error detection system to flag grossly incorrect data for review and to minimize human error.

### Weight Loss and Other Anthropometric Parameters

Body weight was assessed preoperatively and 3, 6, 9, 12, 15, and 24 months postoperatively. Weight loss was reported as percent total weight loss (%TWL), percent excess weight loss (%EWL), and change in body mass index (Δ BMI, kg/m^2^) as stated in the guidelines developed by Brethauer et al. [13]. Fat mass and fat percentage were measured by a bioelectrical impedance analysis (TANITA®), a valid method to determine body composition in obese populations [14]. Sex, age, and height were registered at baseline.

### Physical Activity

Physical activity was assessed preoperatively and 9, 15, and 24 months postoperatively with the Baecke questionnaire [15]. The Baecke questionnaire has been used frequently within studies of the bariatric population [16]. Designed as a 5-point Likert scale, the questionnaire consists of 18 inquiries regarding the amount of time subjects spent on several physical activities. Outcome is expressed in three main subscales: leisure, work, and sport activity. Each subscale reports an index score between 1 and 5, in which a higher score means a higher level of physical activity.

### Cardiorespiratory Fitness

Cardiorespiratory fitness was assessed preoperatively and 9, 15, and 24 months postoperatively. VO_2_max is widely accepted as the best measure for cardiorespiratory fitness, representing the highest amount of oxygen an individual can take in and utilize to produce ATP aerobically while breathing air during heavy exercise [17, 18]. Although generally measured by a maximal exercise test, to avoid medical risks within the morbidly obese population the submaximal Åstrand test (considered a reliable and valid method) was performed to assess VO_2_max [19]. During the Åstrand test, participants cycled for 6 min on a cycle ergometer on submaximal level. After the test, VO_2_max was calculated by combining the patient’s characteristics (age, sex, weight, and fat-free mass) with outcomes of the Åstrand test (level of resistance and heart rate) [20]. Due to equipment limitations, the Åstrand test could not be performed if patients weighed more than 182 kg, or if patients consumed beta blockers or tricyclic antidepressants due to their influence on heart rate. Additionally, some patients were unable to test because of joint problems. Furthermore, the test was discontinued if participants were not able to keep a speed of 50 rpm, or when the patient’s heart rate was insufficient (< 120 or > 170 beats per minute).

Besides as an absolute measure, VO_2_max can also be expressed as VO_2_max relative to weight or relative to fat-free mass (FFM). Since FFM and not body weight is the preferred co-variate for comparing different body size and body composition, VO_2_max relative to fat-free mass (VO_2_max/FFM) was used in the analysis as a measure for cardiorespiratory fitness [21]. This measure best represents the patient’s cardiorespiratory fitness, especially in a bariatric population that changes in body composition, because oxygen is mainly absorbed by muscles and bones and less by fat mass. VO_2_max/FFM was calculated as milliliters oxygen, per kilogram fat-free mass, per minute (ml/kg FFM/min). Additionally VO_2_max/FFM data was separated by sex for comparison to Dutch population reference values [20].

### Statistical Analysis

Statistical analyses were performed using SPSS 23. All continuous variables were visually inspected and tested for normality by the Shapiro-Wilk test. Patients’ characteristics that followed a normal distribution were defined by the mean and standard deviation. Qualitative variables were defined by the number and percentage of cases. Descriptive statistics summarized the patient’s characteristics. Each score per follow-up month was compared to baseline using a paired sample *t* test. In addition, new variables were created to express change between baseline and 24 months: 24 m Δ leisure activity, 24 m Δ work activity, 24 m Δ sport activity, 24 m Δ BMI, and 24 m Δ VO_2_max/FFM. Furthermore, 24 m %TWL and 24 m %EWL were calculated. Multiple regression analysis was performed with change in physical activity (Δ leisure activity, Δ work activity, and Δ sport activity) as independent variables and 24 m %TWL and 24 m Δ VO_2_max/FFM as dependent variables. %TWL was chosen as a representative measure of weight loss, since %TWL is not biased by baseline BMI in the way %EWL can be [22]. Multiple regression analysis adjusted for baseline BMI, age, and sex.

## Results

### Study Population

**Fig. 1:**
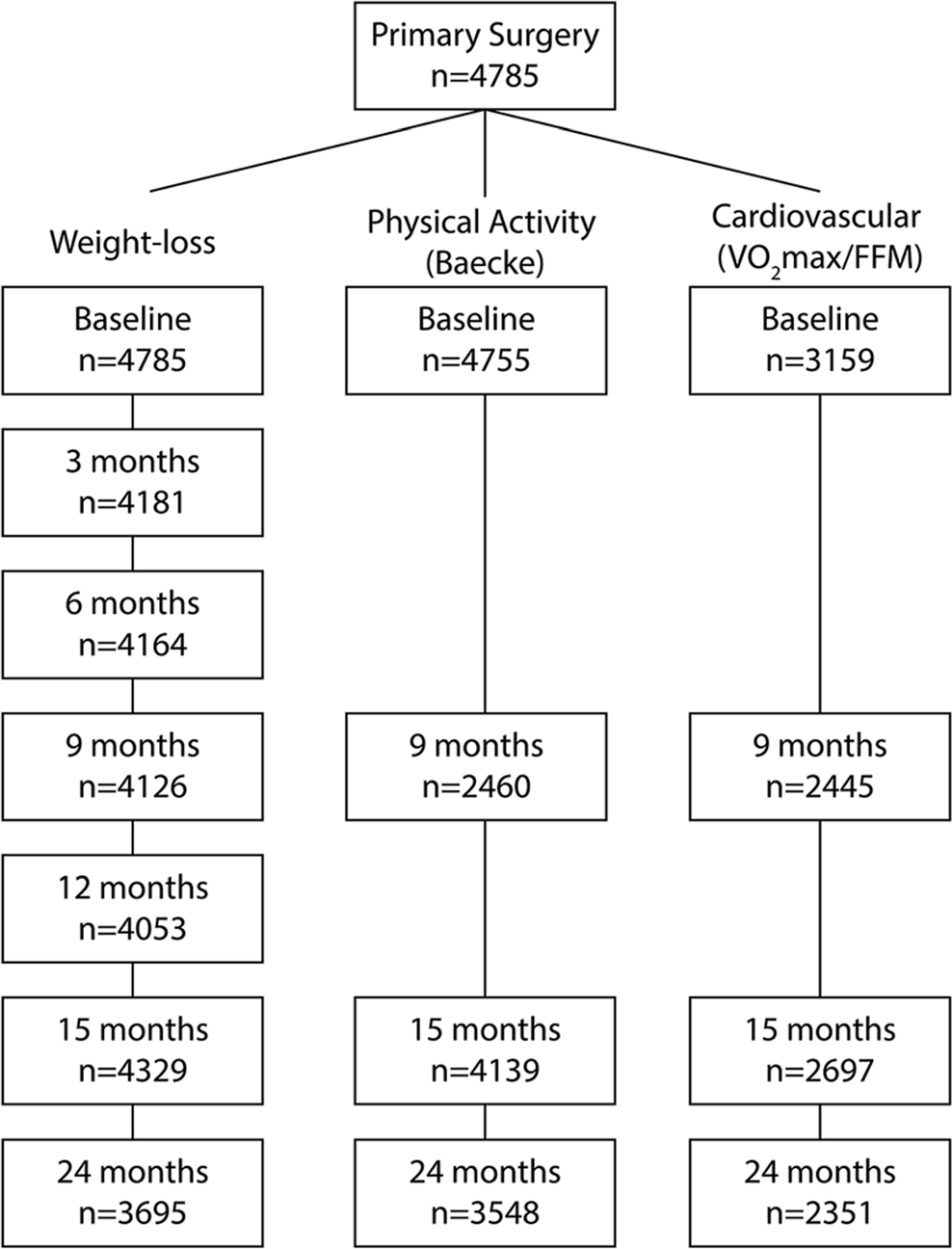
Flow chart of participant follow-up

The study sample consisted of 4785 patients who underwent primary bariatric surgery with a mean age of 43.1 ± 10.7 years, a mean BMI of 44.9 ± 6.2 kg/m^2^ of which 81% were female (Table 1). In 3695 (77%) of the included patients, weight loss data was available at 24 months (Fig. 1). At 24 months, postoperative data was available for the Baecke questionnaire in 3548 (74%) of patients and for the VO_2_max/FFM scores in 2351 (49%) of patients (Fig. 1). The lower availability of VO_2_max/FFM data was due largely to the Åstrand test exclusion criteria (> 182 kg, beta blockers or tricyclic antidepressants, joint problems, and insufficient heartrate (< 120 or > 170)). The median overall compliance of the group sessions (pre- and postoperative) was 70%, with an interquartile range of 60 to 79%.

**Table 1:** Baseline characteristics of the included study population

Total *(n* = 4785)	Mean ± SD	Percentage (*n*)
Age (years)	43.2 ± 10.7	
Female		81% (3867)
BMI (kg/m^2^)	44.9 ± 6.2	
Height (cm)	169.8 ± 8.9	
Weight (kg)	129.7 ± 22	

*BMI*, body mass index

### Physical Activity

**Fig. 2:**
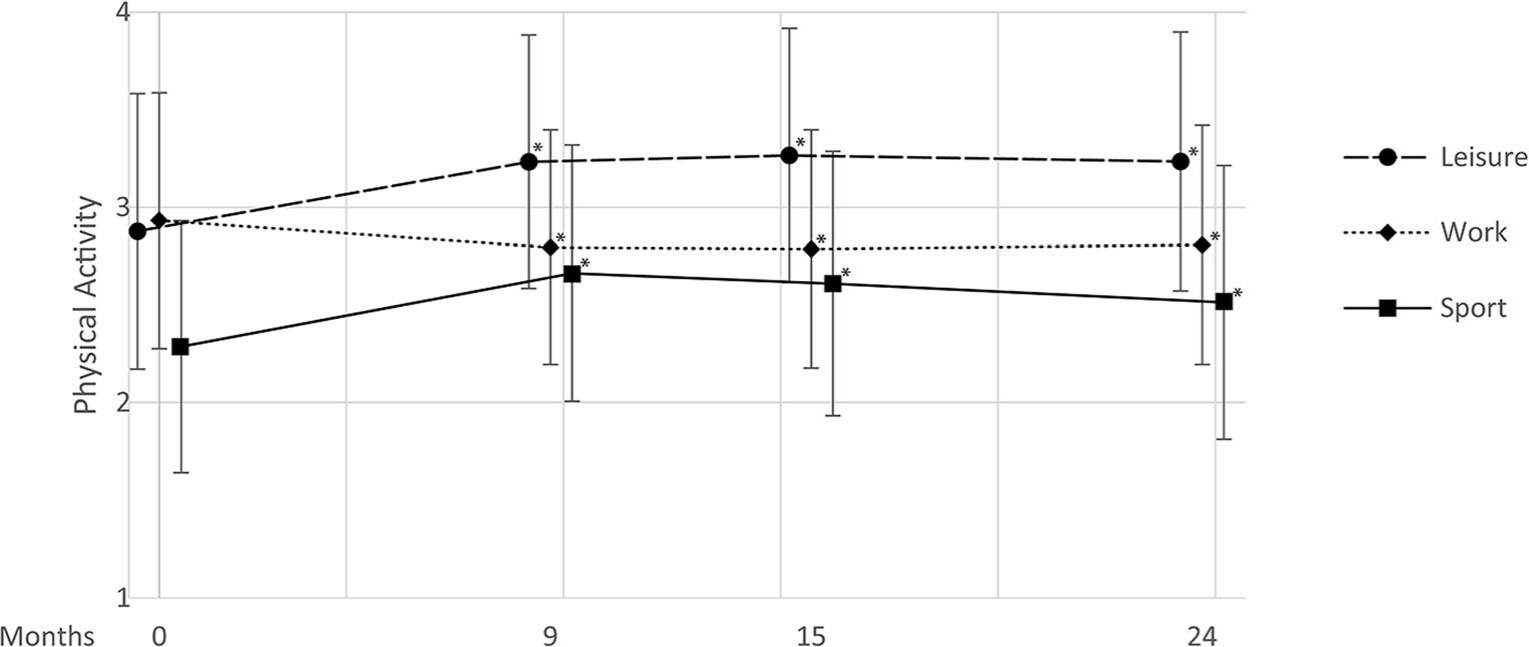
Baseline and follow-up mean (± standard deviation) physical activity scores of Baecke leisure, work, and sport subscales for the entire study population. *P < 0.001 compared to respective baseline; *n* = 2452, 4118, and 3535 at 9, 15, and 24 months, respectively

Baecke questionnaire outcomes showed significant increases in leisure and sport activity at 24 months postoperative compared to baseline (2.88 ± 0.71 vs. 3.23 ± 0.66, *P* < 0.001 and 2.29 ± 0.64 vs. 2.51 ± 0.70, *P* < 0.001, respectively). Furthermore, a small but significant decrease was seen in the work component at 24 months compared to baseline (2.93 ± 0.66 vs. 2.81 ± 0.61, *P* < 0.001). In all cases, significant changes were also seen at earlier 9 and 15 months points when compared to baseline (Fig. 2).

### Weight Loss

**Fig. 3:**
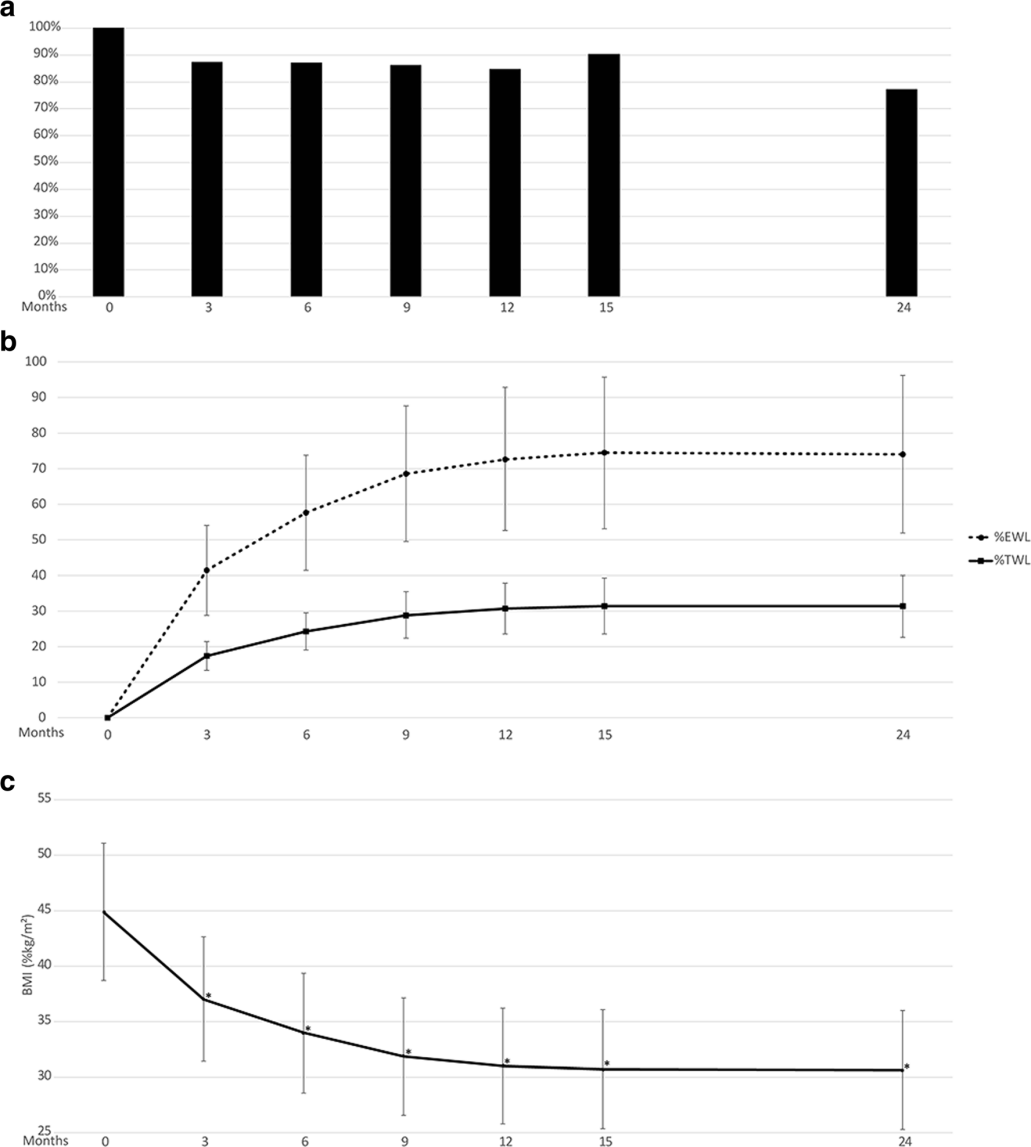
**a** Patient retention for weight loss assessment through 24 months postoperative. **b** %EWL and %TWL through 24 months postoperative. **c** BMI (BMI, kg/m^2^) through 24 months postoperative. *P < 0.001 compared to respective baseline

At the completion of 22 session care program visits within the first 15 months, postoperative patient retention was 91% with a mean TWL of 31.5% (Fig. 3a, b). Follow-up rate at 24 months was 77% with a mean EWL of 74.0% and mean TWL of 31.3% (Fig. 3a, b). Significantly reduced BMI was also reported at each follow-up month throughout the 24 months when compared to baseline (Fig. 3c).

### Cardiorespiratory Fitness

**Fig. 4:**
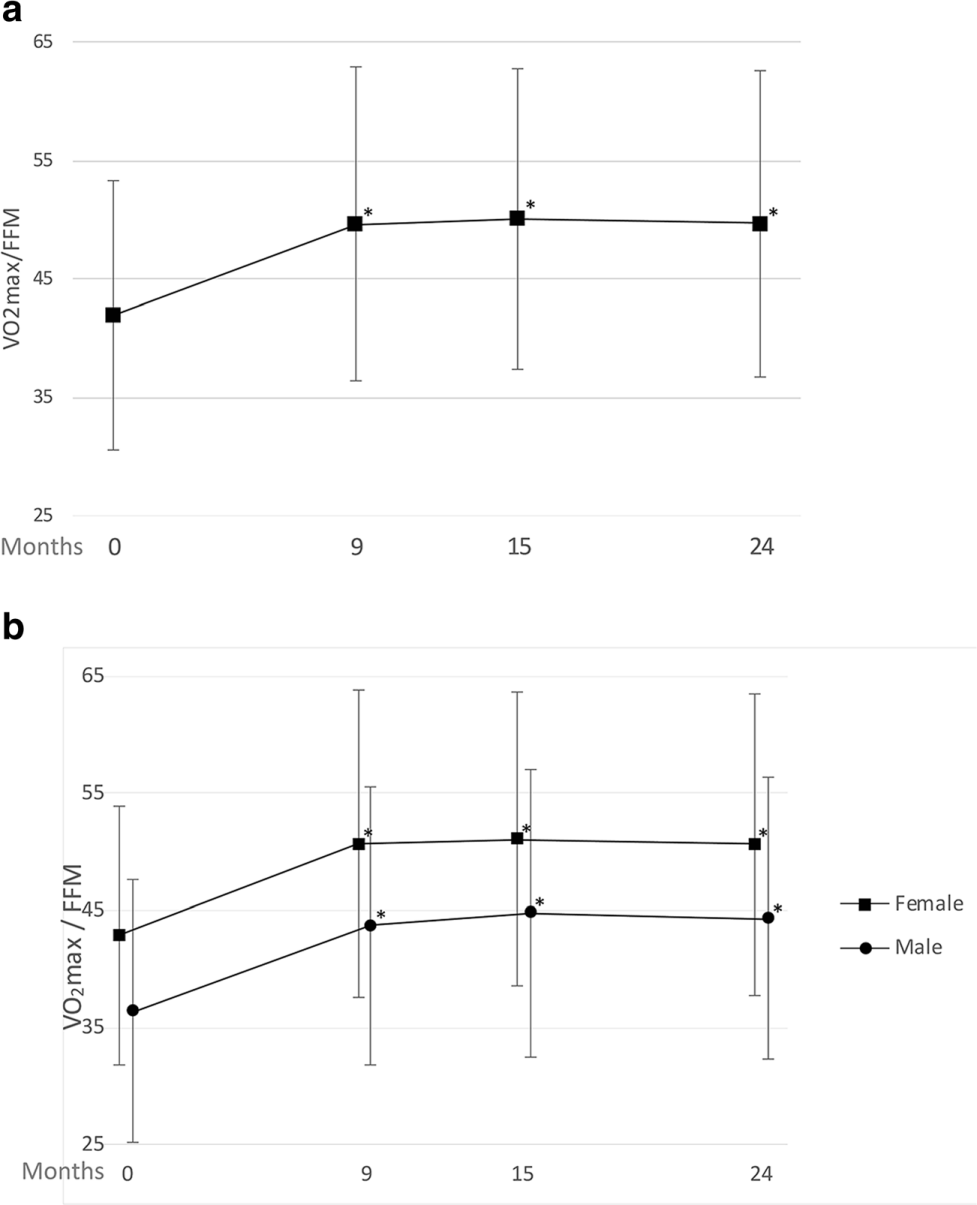
**a** Baseline and follow-up mean (± standard deviation) Åstrand bicycle test outcomes, showing VO_2_Max/FFM (ml/kg FFM/min). *P < 0.001 compared to baseline; *n* = 1900, 2072, and 1825 at 9, 15, and 24 months respectively. **b** VO_2_Max/FFM from females (81%) and males (19%). *P < 0.001 compared to respective baseline

Åstrand bicycle test outcomes showed significant increase of mean absolute VO_2_max between baseline and 24 months follow-up (3.05 ± 0.79 vs. 3.25 ± 0.88, *P* < 0.001). Cardiorespiratory fitness, VO_2_max/FFM, also improved significantly throughout the study with an increase from 41.94 ± 11.32 at baseline to 49.67 ± 12.95 at 24-months (*P* < 0.001). Significant improvement in VO_2_max/FFM was also seen at each follow-up month throughout the 24 months when compared to baseline (Fig. 4a). When separated by sex, male patients showed a lower cardiorespiratory fitness through the 24-month period, although the overall change between baseline and 24 months was similar when comparing males and females in both groups (Fig. 4b). For both males and females, VO2max/FFM at 9, 15, and 24 months showed significant increases when compared to their respective baselines.

### Association of Physical Activity with Weight Loss and Cardiorespiratory Fitness

Change in leisure activity at 24 months was positively associated with %TWL at 24 months (*P* < 0.001, Table 2), thus patients who had higher improvements in leisure activity had better weight loss. No significant associations of change in sport and work activity were found with %TWL however.

**Table 2:** Multiple regression analyses of change in physical activity relating to %TWL and change in VO_2_max/FFM

Independent variable	Dependent variable	*N*	*ß*	95% CI		*p* value	*R* ^*2*^
				Lower	Upper		
Δ sport Δ leisure Δ work	24 m %TWL	3484				> .05	
	24 m %TWL	3484	1.352	.912	1.792	< .001	.054
	24 m %TWL	3484				> .05	
Δ sport Δ leisure Δ work	24 m Δ VO_2_max/FFM	1743	1.730	1.039	2.420	< .001	.070
	24 m Δ VO_2_max/FFM	1743				> .05	
	24 m Δ VO_2_max/FFM	1743				> .05	

24 m *%TWL*, percentage total weight loss at 24-month follow-up

24 m Δ *VO*_*2*_*max/FFM*, change in VO_2_max relative to fat-free mass (ml/kg FFM/min) at 24-month follow-up

Multiple regression analysis was adjusted for baseline BMI, age, and sex

Change in sport activity at 24 months was positively associated with change in VO_2_max/FFM at 24 months (*P* < 0.001, Table 2), thus patients who had higher improvements in sport activity had better cardiorespiratory fitness improvements. No significant associations of change in leisure and work activity with change in VO_2_max/FFM were identified.

## Discussion

The aim of this study was to assess changes in physical activity, weight loss, and cardiorespiratory fitness after bariatric surgery complemented by a perioperative lifestyle change program and to evaluate the association of changes in physical activity with weight loss and change in cardiorespiratory fitness. Results showed significant postoperative improvement in physical activity, weight loss, and cardiorespiratory fitness. In addition, positive associations were found between change in sport activity and change in cardiorespiratory fitness, but not weight loss. While on the other hand, positive associations were found between change in leisure activity and weight loss, but not change in cardiorespiratory fitness.

A positive aspect of this study is the robustness of the sample, resulting in large statistical power. However, this also increases the chance on finding significant effects that may not be clinically relevant. Nevertheless, the observed improvements in health outcomes from preoperative to 2 years postoperative have a substantial impact on the patient’s life. For example, the EWL of 74% reported in this study is higher than the weight loss results seen in the systematic review of bariatric clinical studies by Puzziferri et al. in 2014 [23]. Furthermore, improvements in VO2max are associated with lower risks of all-cause and cardiovascular mortality [24]. In this study, cardiorespiratory fitness as measured by VO2max relative to fat-free mass improved by 18.4% compared to pre-surgery measures (41.94 to 49.67 respectively), reflecting a substantial improvement. In comparison, most training studies (3–12 months) in patients achieve improvements of less than 10–15% [25].

To better understand the clinical relevance of the improvement in cardiorespiratory fitness, Dutch VO_2_max/FFM reference values of a healthy trained and a healthy untrained male and female population were evaluated [20]. When compared to these values, male patients showed a much lower VO_2_max/FFM at baseline as well as at 24 months (36.4 and 44.3, respectively) than the trained or untrained healthy male population (48.5 and 55.2, respectively). In contrast, female patients showed just a slightly lower VO_2_max/FFM at baseline than the untrained healthy female population and at 24 months their VO_2_max/FFM was almost as high as the trained healthy female population (female patients at baseline and 24 months, 42.9 and 50.7, respectively; healthy untrained and healthy trained female population, 44.0 and 52.7, respectively). Although VO_2_max/FFM between male and female patients was different, both showed similar improvements of 7.9 and 7.8 ml/kg FFM/min, respectively. These improvements between baseline and 24 months in VO2max/FFM are similar to the differences between the healthy untrained and healthy trained Dutch populations for both men and women (6.7 and 8.7 ml/kg FFM/min, respectively), indicating a clinically relevant level of cardiorespiratory fitness improvement.

It is not completely clear why the male patients have lower cardiorespiratory fitness than the female patients, both at baseline and postoperative. A higher BMI and more comorbidities in male patients pre- and postoperative may explain the lower cardiorespiratory fitness in men compared to women. The low preoperative VO_2_max in male patients indicates that male patients specifically may benefit from an improvement in VO_2_max, as low VO_2_max is associated with cardiovascular disease and strongly predicts one’s risk of dying from heart attack or stroke [26, 27].

The positive association of change in sport activity with change in cardiorespiratory fitness and not with weight loss, and change in leisure activity with weight loss and not with change in cardiorespiratory fitness, may be explained by the nature of the activities. Since sport requires a relatively high level of activity and leisure activity occurs relatively frequent, activity in sport might be more likely to increase cardiorespiratory fitness, while the more frequent leisure activity might help burn more calories over a long-time period, resulting in higher weight loss.

The results of this study demonstrate that bariatric surgery complemented by a comprehensive pre- and postoperative bariatric care program have the potential to increase physical activity, weight loss, and cardiorespiratory fitness. As previous studies have shown, this can reduce cardiovascular diseases and mortality as well as potentially decrease health care costs [8, 28]. Therefore, this treatment might have substantial beneficial consequences for the patients involved. Furthermore, the associations of physical activity with cardiorespiratory fitness and weight loss that were found suggest that practitioners could influence the patient’s outcome by stimulating sport and leisure activity. Recognition of these factors could aid in tailoring an intensive support program to improve clinical results.

Our analysis was limited by the availability of VO_2_max/FFM data, which was lower than that of Baecke and weight loss data. This was largely due to the exclusion criteria of the Åstrand test (> 182 kg, beta blockers or tricyclic antidepressants, joint or knee problems, insufficient heartrate during test). Since the absolute number of patients with available VO_2_max/FFM data was more than adequate, the association of change in physical activity with change in VO_2_max/FFM could still be demonstrated. For patients who are unable to complete an Åstrand test (e.g., not able to ride a stationary bicycle or weigh > 182 kg), we recommend considering the Bruce Protocol as an alternative instrument to measure VO_2_max [29, 30]. Additionally, although the Baecke is a self-reported questionnaire and may suffer from over- or underreporting, due to the retrospective design in this study, we think that these influences would minimally affect the overall scores. Moreover, if any bias occurred, we expect it to affect both preoperative and postoperative measures and therefore have minimal influence on Baecke change scores. However, future studies may wish to include quantifiable measures of physical activity to obtain more insight into physical activity patterns. Finally, there are differences in ability to improve VO_2_max/FFM between patients. For example, lower weight individuals may be able to improve less than overweight individuals, resulting in smaller but still substantial improvements. Additional research to assess these groups could provide insight into treatments based on the severity of individual weight.

## Conclusion

In conclusion, this study demonstrated significant increase in physical activity, weight loss, and cardiorespiratory fitness after bariatric surgery complemented by a comprehensive pre- and postoperative lifestyle change program. Furthermore, improvement in sport activity was associated with improvement in cardiorespiratory fitness and improvement in leisure activity was associated with higher weight loss. By incorporating physical activity, bariatric programs could enhance weight loss and cardiorespiratory fitness to potentially reduce cardiovascular disease, mortality, and health care costs.
